# Average and statistical properties of coherent radiation from steady-state microbunching

**DOI:** 10.1107/S1600577522009973

**Published:** 2023-01-01

**Authors:** X. J. Deng, Y. Zhang, Z. L. Pan, Z. Z. Li, J. H. Bian, C.-Y. Tsai, R. K. Li, A. W. Chao, W. H. Huang, C. X. Tang

**Affiliations:** a Department of Engineering Physics, Tsinghua University, Beijing 100084, People’s Republic of China; b School of Electrical and Electronic Engineering, Huazhong University of Science and Technology, Wuhan 430074, People’s Republic of China; c Institute for Advanced Study, Tsinghua University, Beijing 100084, People’s Republic of China; d Stanford University, Stanford, CA 94309, USA; NSRRC, Taiwan

**Keywords:** accelerator light source, steady-state microbunching, transverse form factor, radiation fluctuation, EUV lithography, ARPES, coherent radiation

## Abstract

A promising accelerator light source mechanism called steady-state microbunching (SSMB) is being actively studied. With the combination of strong coherent radiation from microbunching and high repetition rate of a storage ring, high-average-power narrow-band radiation can be anticipated from an SSMB storage ring, with wavelengths ranging from THz to soft X-ray. Such a novel light source could provide new opportunities for accelerator photon science like high-resolution angle-resolved photoemission spectroscopy and industrial applications like extreme ultraviolet (EUV) lithography. In this paper, a theoretical and numerical study of the average and statistical properties of coherent radiation from SSMB are presented. The results show that 1 kW average-power quasi-continuous-wave EUV radiation can be obtained from an SSMB ring provided that an average current of 1 A and a microbunch train with bunch length of 3 nm can be formed at the radiator which is assumed to be an undulator. Together with the narrow-band feature, the EUV photon flux can reach 6 × 10^15^ photons s^−1^ within a 0.1 meV energy bandwidth, which is three orders of magnitude higher than that in a conventional synchrotron source and is appealing for fundamental condensed matter physics and other research. In this theoretical investigation, we have generalized the definition and derivation of the transverse form factor of an electron beam which can quantify the impact of its transverse size on coherent radiation. In particular, it has been shown that the narrow-band feature of SSMB radiation is strongly correlated with the finite transverse electron beam size. Considering the pointlike nature of electrons and quantum nature of radiation, the coherent radiation fluctuates from microbunch to microbunch, or for a single microbunch from turn to turn. Some important results concerning the statistical properties of SSMB radiation are presented, with a brief discussion on its potential applications, for example the beam diagnostics. The presented work is of value for the development of SSMB to better serve potential synchrotron radiation users. In addition, this also sheds light on understanding the radiation characteristics of free-electron lasers, coherent harmonic generation, *etc*.

## Abstract 2

The radiation properties of a novel high-power accelerator light source are presented. Potential applications include high-resolution angle-resolved photoemission spectroscopy and extreme ultraviolet lithography.

## 1. Introduction

Particle accelerators as photon sources are advanced tools in investigating the structure and dynamical properties of matter, and have enabled advances in science and technology for more than half a century (Chao & Chou, 2011 ▸). The present workhorses of these sources are storage-ring-based synchrotron radiation facilities (Elder *et al.*, 1947 ▸; Tzu, 1948 ▸; Schwinger, 1949 ▸; Zhao, 2010 ▸) and linear-accelerator-based free-electron lasers (FELs) (Madey, 1971 ▸; Kondratenko & Saldin, 1980 ▸; Bonifacio *et al.*, 1984 ▸; Emma *et al.*, 2010 ▸; Huang & Kim, 2007 ▸; Pellegrini *et al.*, 2016 ▸). These two kinds of sources deliver light with high repetition rate and high peak brilliance and power, respectively. Some applications, however, do need high average power and high photon flux. Kilowatt extreme ultraviolet (EUV) light sources, for example, are urgently needed by the semiconductor industry for EUV lithography (Bakshi, 2018 ▸). Ultrahigh-energy-resolution angle-resolved photoemission spectroscopy (ARPES) requires that the initial photon flux before the monochromator is sufficiently high (Damascelli *et al.*, 2003 ▸). To realize high average power and photon flux, a high repetition rate or a high peak power alone is not sufficient. We need both of them simultaneously.

The key to the high peak power of FELs lies in microbunching, which means the electrons are bunched or sub-bunched to a longitudinal dimension smaller than the radiation wavelength so that the electrons radiate in phase and thus cohere (Schwinger, 1996 ▸; Nodvick & Saxon, 1954 ▸; Gover *et al.*, 2019 ▸). The power of coherent radiation is proportional to the number of radiating electrons squared, and therefore can be orders of magnitude stronger than the equivalent incoherent radiation in which the power dependence on the electron number is linear. The self-amplified spontaneous emission (SASE) scheme (Kondratenko & Saldin, 1980 ▸; Bonifacio *et al.*, 1984 ▸) of microbunching making high-gain FELs extremely powerful is actually, however, a collective beam instability which degrades the electron beam parameters, and the microbunching can only be exploited once. The repetition rate of the radiation is thus limited by the repetition rate of the driving source, *i.e.* the linear accelerator. In addition, as the FEL radiation in SASE originates from the shot noise of the electron beam, it has a large shot-to-shot power fluctuation and noisy energy spectrum. There are now active efforts devoted to high-repetition-rate FELs, for example the superconducting FEL (Altarelli *et al.*, 2007 ▸; Galayda, 2018 ▸; Zhu *et al.*, 2017 ▸), X-ray FEL oscillator (Kim *et al.*, 2008 ▸), energy-recovery linac (ERL) (Nakamura, 2012 ▸) and similar. Active efforts are also ongoing concerning seeded FELs to improve the temporal coherence and stability of FEL radiation (Yu, 1991 ▸; Stupakov, 2009 ▸; Hemsing *et al.*, 2014 ▸). However, the realization of a high-average-power, narrow-band, stable continuous-wave (CW) short-wavelength light source remains a challenge.

A mechanism called steady-state microbunching (SSMB) was proposed by Ratner & Chao (2010 ▸) to resolve this issue. The idea of SSMB is that, by phase-space manipulation of an electron beam, microbunching forms and stays in a steady state each time it travels through a radiator in a storage ring. The steady state here means a balance of quantum excitation and radiation damping, a true equilibrium in the context of storage-ring beam dynamics. Once realized, the strong coherent radiation from microbunching and the high repetition rate of a storage ring combine to form a high-average-power photon source.

**Figure 1:**
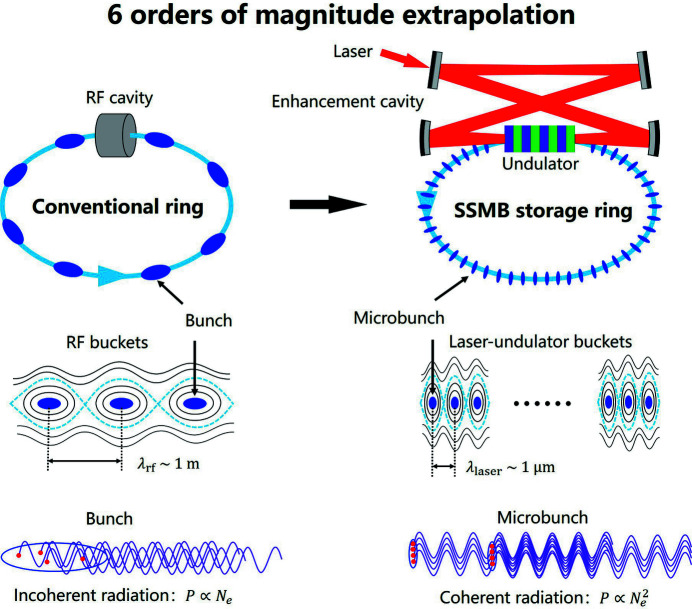
Schematic layout of a conventional storage ring (left) and an SSMB storage ring (right).

A schematic layout of an SSMB storage ring and its operating principle in comparison with a conventional storage ring are shown in Fig. 1 ▸. SSMB replaces the conventional bunching system in a storage ring, namely the radiofrequency (RF) cavity, with a laser modulation system. As the wavelength of a laser (∼µm) is typically six orders of magnitude smaller than that of an RF wave (∼m), a much shorter bunch, *i.e.* a microbunch, can thus be anticipated by invoking this replacement together with a dedicated magnetic lattice. To provide adequate and stable longitudinal focusing such that microbunches can be formed and sustained, SSMB requires a powerful phase-locked laser to interact with electrons on a turn-by-turn basis. The realization of such a laser system usually demands an optical enhancement cavity. A laser cannot effectively interact with the co-propagating electrons if the electrons travel in a straight line, as the electric field of a laser is perpendicular to the laser propagation direction. A modulator which bends the electron trajectory transversely is thus needed. The modulator is usually an undulator, which is a periodic structure of dipole magnets. Note that to avoid head-on collisions (Compton backscattering) between the reflected laser and the electrons, a four-mirror optical cavity, instead of a two-mirror one, is chosen for the illustration in Fig. 1 ▸.

The microbunching in SSMB is a result of the active longitudinal focusing provided by the laser modulator, similar to conventional RF bunching through the phase stability principle (Veksler, 1944 ▸; McMillan, 1945 ▸). The radiation in SSMB, unlike that in a FEL, is a passive process and the radiator can be rather short, for example it can be a simple dipole magnet or a short undulator. The modulator undulator is also much shorter than the radiator undulator in a high-gain FEL. Therefore, there is no FEL mechanism invoked in the bunching or radiation process in SSMB. Any unavoidable FEL effects need to be controlled within a safe region so that the steady state micobunches are not destroyed.

Note that we have not presented explicitly the energy compensation system for SSMB in Fig. 1 ▸. The modulation laser in principle can be used to supplement the radiation energy loss of the electrons, just like the traditional RF, but this may not be a cost-effective choice. Besides, the electron beam current and output radiation power will also be limited by the incident laser power. Instead, one may just use a traditional RF cavity for the energy compensation. If a larger filling factor of the electron beam is desired, it could also be one or several induction linacs. In the present envisioned high-average-power SSMB photon source, an induction linac is tentatively used as the energy compensation system and the filling factor of the electron beam in the storage ring can be rather large, for example larger than 50%. The radiation waveform of such an SSMB light source is thus CW or quasi-CW, if radiation from different microbunches connects with each other and forms a continuous long pulse.

The potential of SSMB as a new light source mechanism is tremendous, as can be viewed from two perspectives.

(i) From the accelerator physics perspective, there is now active and important development on low-transverse-emittance or diffraction-limited storage rings (Teng, 1984 ▸; Eriksson *et al.*, 2014 ▸), which focuses on the transverse dimension of the electron beam. SSMB, however, is being used to investigate the potential of the longitudinal dimension of the electron beam in storage rings. The large compactification of the bunching system wavelength (from metres to micrometres) provides scope for exciting developments in accelerator physics.

(ii) From the synchrotron radiation application perspective (Deng *et al.*, 2021*a* ▸), once realized, SSMB can deliver photons with high average power, high repetition rate (MHz to CW) and narrow bandwidth, at wavelengths ranging from THz to soft X-ray. Such a novel photon source could provide unprecedented opportunities for accelerator photon science and technological applications. For example, SSMB is promising for generating high-power EUV radiation for EUV lithography (Bakshi, 2018 ▸). Energy-tunable high-flux narrow-band EUV photons are also highly desirable in condensed matter physics studies, such as high-resolution ARPES (Damascelli *et al.*, 2003 ▸; Lv *et al.*, 2019 ▸). Ultrahigh-power deep ultraviolet and infrared sources are potential research tools in atomic and molecular physics. Moreover, new nonlinear phenomena and dynamical properties of materials can be driven and studied by high-peak and average-power THz sources (Carr *et al.*, 2002 ▸; Cole *et al.*, 2001 ▸). Besides high power, SSMB can also produce ultrashort (sub-femtosecond to attosecond) photon pulse trains with definite phase relations, which could be useful in attosecond physics investigations (Krausz & Ivanov, 2009 ▸).

To promote SSMB research and in particular develop an EUV SSMB storage ring, a task force has been established at Tsinghua University (Tang *et al.*, 2018 ▸; Pan *et al.*, 2019 ▸; Deng *et al.*, 2020*a* ▸,*b* ▸, 2021*a* ▸,*b* ▸,*c* ▸; Zhang *et al.*, 2021 ▸; Tang & Deng, 2022 ▸; Deng, 2022 ▸), in collaboration with researchers from other institutes. A key progress of the collaboration is the recent success of the SSMB proof-of-principle experiment conducted at the Metrology Light Source (MLS) in Berlin, Germany (Deng *et al.*, 2021*a* ▸). The SSMB beam physics study (Deng *et al.*, 2020*a* ▸,*b* ▸, 2021*b* ▸,*c* ▸; Zhang *et al.*, 2021 ▸; Deng, 2022 ▸) and magnet lattice design (Pan *et al.*, 2019 ▸) for the EUV SSMB storage ring are also ongoing, with encouraging progress being achieved. Other active SSMB-related research activities are also ongoing (Jiao *et al.*, 2011 ▸; Chao *et al.*, 2016 ▸; Khan, 2017 ▸; Li *et al.*, 2019 ▸; Tsai *et al.*, 2021 ▸; Tsai, 2022*a* ▸,*b* ▸; Zhao *et al.*, 2021 ▸; Lu *et al.*, 2022 ▸).

In parallel to the above-mentioned efforts, for a better service to the user community, the SSMB radiation characteristics and their dependence on beam and radiator parameters need to be investigated. An in-depth understanding of the impact of radiation on the electron beam is also of vital importance for the stable operation of SSMB. In addition, rich information about the electron beam is embedded in the radiation characteristics. Therefore, the radiation can also be a useful beam diagnostics tool. This is the motivation for us to study the SSMB radiation in this work.

The contents of this paper are organized as follows. In Section 2 we present the general formulation of coherent radiation from a three-dimensional rigid beam. The longitudinal and transverse form factors of an electron beam are introduced in Section 3 to quantify the impact of its longitudinal and transverse distribution on coherent radiation. The analytical expressions of the transverse form factor for the interested undulator radiation is derived and benchmarked with direct numerical integration. The implications of the derived form factor are also discussed. Based on the form factors, the coherent radiation power and spectral flux at harmonics of the undulator radiation are derived in Section 4. The statistical characteristics of the radiation are analyzed in Section 5, with a brief discussion on its potential applications. An example calculation for the envisioned EUV SSMB radiation is presented in Section 6, and a short summary is given in Section 7.

## 2. Formulation of radiation from a rigid beam

For simplicity, in this paper we adopt the rigid beam approximation. More specifically, we consider only the impact of particle position *x*, *y* and *z*, but ignore the particle angular divergence *x*′, *y*′ and energy deviation δ, on the radiation. Under this approximation, concise and useful analytical formulas for the coherent radiation can be obtained. This approximation is accurate when the transverse and longitudinal beam size do not change much inside the radiator, *i.e.* β_*x*,*y*_ ≳ *L*_r_ and β_*z*_ ≳ *R*_56,r_, where β_*x*,*y*,*z*_ are the Courant–Snyder functions of the beam in the horizontal, vertical and longitudinal dimensions (Courant & Snyder, 1958 ▸), *L*_r_ and *R*_56,r_ are the length and momentum compaction of the radiator, respectively. When this is not the case, applying the average beam size inside the radiator still gives a reasonable result. We will see later in Section 6 that the conditions of the rigid beam approximation are generally satisfied in the envisioned EUV SSMB.

**Figure 2:**
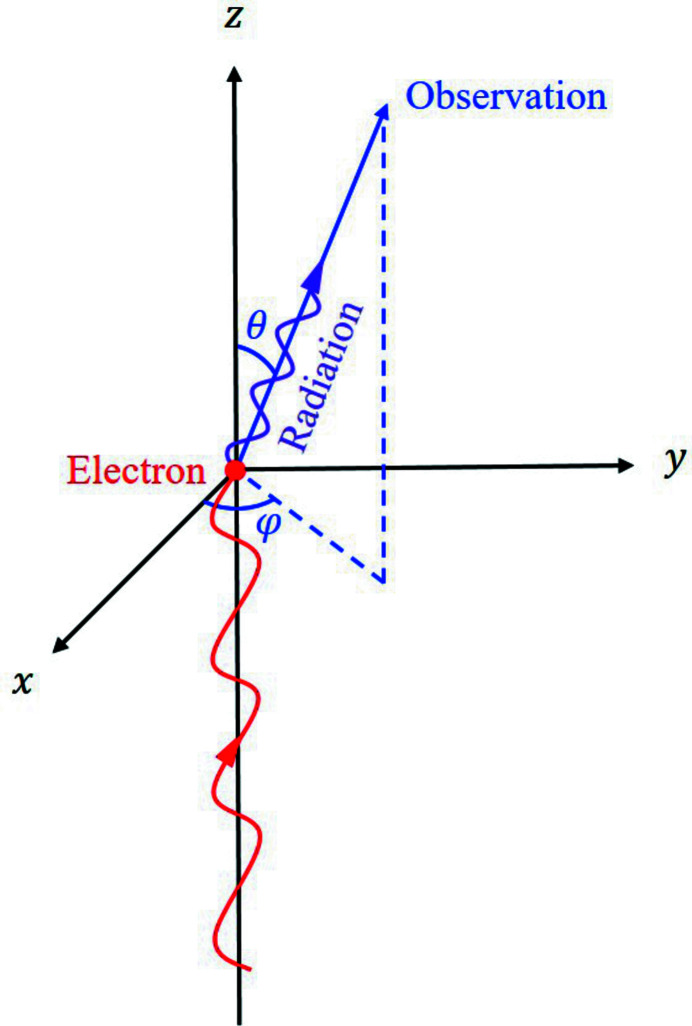
Coordinate system used to calculate the undulator radiation spectrum. The magnetic field is in the *y*-direction, and the electron wiggles in the *x*–*z* plane.

Assume that the radiation vector potential of the reference particle at the observation location is **A**_point_(θ, φ, *t*), with θ and φ being the polar and azimuthal angles in a spherical coordinate system, respectively, as shown in Fig. 2 ▸. Under the far-field approximation, the vector potential of a three-dimensional (3D) rigid electron beam containing *N*_e_ electrons is then 

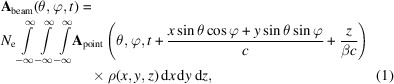

in which β is the particle velocity normalized by the speed of light in a vacuum *c*, and ρ(*x*, *y*, *z*) is the normalized charge density satisfying 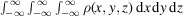 = 1. Note that we have assumed that the particle motion pattern, and therefore also the radiation pattern of a single electron, does not depend on *x*, *y*, *z* of the particle. In other words, *x*, *y*, *z* of a particle only influence the arrival time of the radiation at the observation. This is the reason why their impacts can be treated within a single framework. The impacts of *x*′, *y*′ and δ are different. Generally, their impacts are twofold. First, they affect the radiation of the single particle itself, *i.e.* the radiation pattern. Second, they affect the electron beam distribution, therefore the coherence of different particles, during the radiation process. In this paper, we focus on the case of a ‘3D rigid’ beam.

According to the convolution theorem, for a 3D rigid beam, we now have 

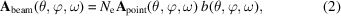

where 

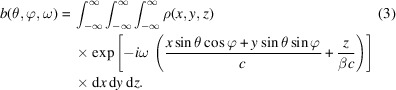

Since **A**(θ, φ, *t*) is real, then **A**(θ, φ, −ω) = **A** ^*^(θ, φ, ω). The energy radiated per unit solid angle per unit frequency interval is (Jackson, 1999 ▸) 

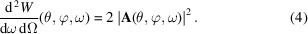

Therefore, we have 

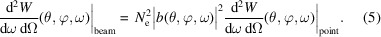

The total radiation energy spectrum of a beam can be obtained by integration with respect to the solid angle, 

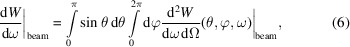

and the total radiation energy of the beam can be calculated by further integration with respect to the frequency, 

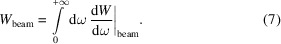

The reason why the lower limit in the above integration is 0, instead of −∞, is that there is a factor of two in the definition of equation (4). The above formulas can be used to numerically calculate the characteristics of radiation from an electron beam, once its 3D distribution is given. Note that in the relativistic case we only need to account for θ within a range of several times of 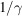 , as the radiation is very collimated in the forward direction.

## 3. Form factors

When the longitudinal and transverse dimensions of the electron beam are decoupled, we can factorize *b*(θ, φ, ω) as 

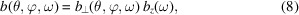

where 

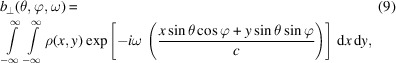

and 

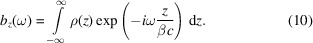

Note that ρ(*x*, *y*) and ρ(*z*) are then the projected charge density. *b*_*z*_(ω) is the usual bunching factor found in the literature and is independent of the observation angle. This, however, is not true for 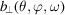 . For example, in the case of a 3D Gaussian *x*–*y*–*z* decoupled beam,

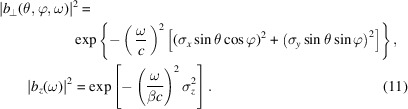

In order to efficiently quantify the impact of the transverse and longitudinal distributions of an electron beam on the overall radiation energy spectrum, here we define the transverse and longitudinal form factors of an electron beam as

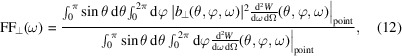

and 

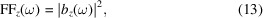

respectively. The overall form factor is then 

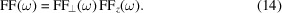

The total radiation energy spectrum of a beam is related to that of a single electron by 

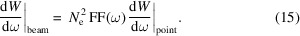

The longitudinal form factor is independent of the radiation process and has been discussed extensively in the literature, so now let us focus on the transverse form factor. Since the transverse form factor depends on the radiation process, there is not a universal formula involving only the beam distribution. Here, for our interest, we investigate the case of undulator radiation. We use a planar undulator as an example. The formulation for a helical undulator is similar.

As is well established in the literature, the planar undulator radiation of a point charge in the *H*th harmonic is (Chao, 2020 ▸) 

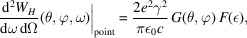




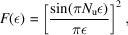




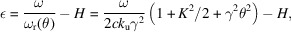




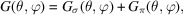




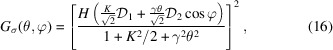




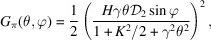




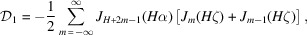




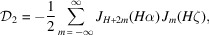




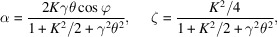

in which *e* is the elementary charge, γ is the Lorentz factor, ε_0_ is the permittivity of free space, ω_r_(θ) is the fundamental resonant angular frequency at the observation with a polar angle of θ, *k*_u_ = λ_u_/2π is the wavenumber of the undulator, *K* = 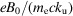 = 0.934*B*_0_[T]λ_u_[cm] is the undulator parameter, with *B*_0_ being the peak magnetic field strength of the undulator and *m*_e_ being the mass of an electron, and *J* being the Bessel function.

Now we try to obtain some analytical results for the transverse form factor. The motivation is that these analytical results can help us better understand the radiation physics and give us an efficient evaluation of the radiation characteristics. Instead of a general discussion, here we only consider the simplest case of a transverse Gaussian round beam, *i.e.*


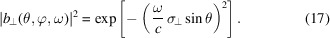

As the radiation is dominantly in the forward direction in the relativistic case, and 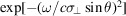 approaches zero with the increase of θ, therefore in equation (12) the upper limit of θ in the integration can be effectively replaced by infinity, and 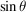 can be replaced by θ in 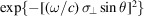 . Then 

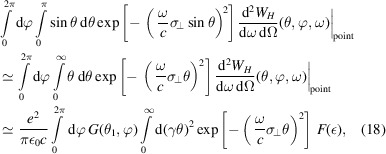

where 

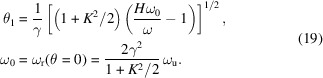

Here we have made use of the fact that there is only one value of θ, *i.e.* θ_1_, that contributes significantly to the integration over the solid angle Ω due to the sharpness of *F*(ε) when the undulator period number *N*_u_ ≫ 1, as the spectral width of *F*(ε) is 1/*N*_u_.

The transverse form factor corresponding to the *H*th harmonic can thus be defined as 

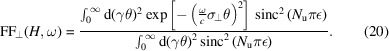

The radiation spectrum of the *H*th harmonic is then 

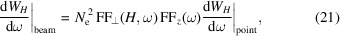

and the total radiation spectrum of an electron beam is 

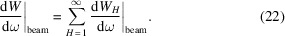

Denote 

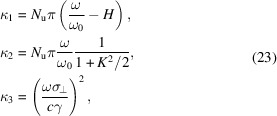

then the denominator in equation (20) is 

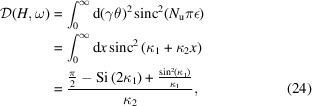

where 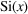 = 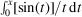 is the sine integral, and the numerator in equation (20) is 

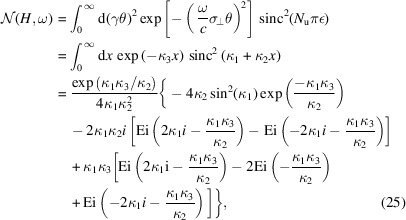

where Ei(*x*) = 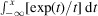 is the exponential integral. The transverse form factor is then 

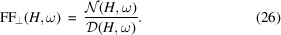

When ω = *H*ω_0_, then κ_1_ = 0, we have 

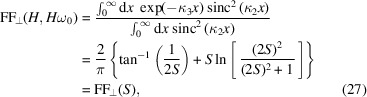

where 

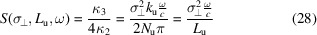

is the diffraction parameter, with *L*_u_ = *N*_u_λ_u_ being the length of the undulator. This form factor 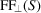 is a universal function and has been obtained before (Saldin *et al.*, 2005 ▸). Here we have reproduced the result following the general definition of the transverse form factor. The variable *S* is a parameter used to classify the diffraction limit of the beam, 

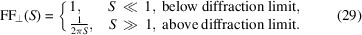

**Figure 3:**
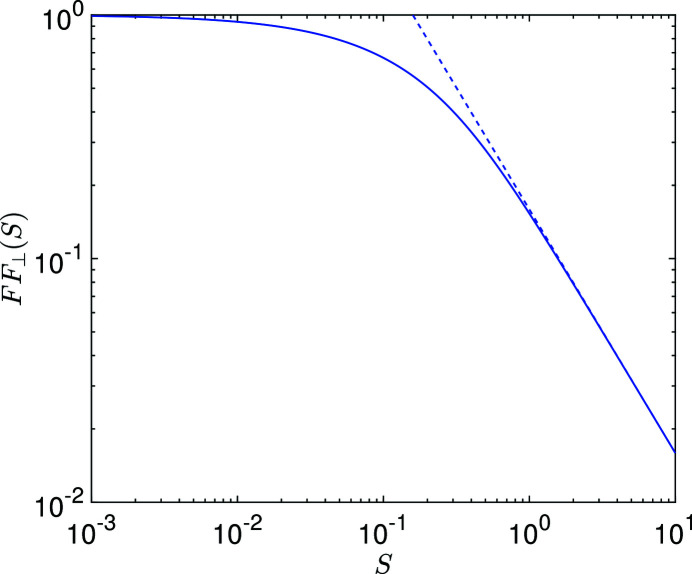
The universal function 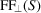 and its asymptotic value above the diffraction limit. The solid line comes from equation (27), the dashed line from the asymptotic relation above the diffraction limit equation (29).

This function, along with its asymptotic result above the diffraction limit, is shown in Fig. 3 ▸.

Note that the decrease of 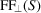 with the increase of 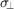 (as 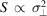 ) means that the coherent radiation at the frequency ω = *H*ω_0_ becomes less when the transverse electron beam size becomes larger. This reflects the fact that for a given radiation frequency ω there are a range of polar angles θ that can contribute. For ω = *H*ω_0_, not only θ = 0 but also θ very close to 0 contribute. With the increase of 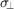 , the effective bunching factor *b*(θ, φ, ω) at ω = *H*ω_0_ drops for these non-zero θ due to the projected bunch lengthening, and therefore the coherent radiation becomes less. Another way to appreciate the drop of 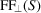 with the increase of 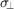 is that there is a transverse coherence area whose radius is proportional to (*L*_u_λ_0_/*H*)^1/2^ with λ_0_ = 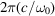 , and fewer particles can cohere with each other when the transverse size of the electron beam increases.

Note that our definition equation (12) and derivation of the transverse form factor equation (26) is more general than that given by Saldin *et al.* (2005 ▸), as they cover other frequencies in addition to a single frequency ω_0_. Therefore, it contains more information than equation (27) as will be presented soon. The issue of equation (26) is that it is still not simple enough for analytical evaluation to provide physical insight. A further approximation is thus introduced, 

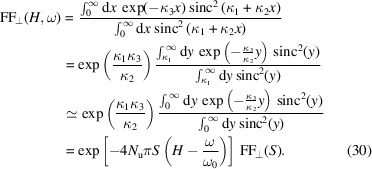

The condition of applying such a simplification is 

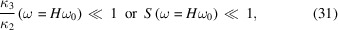


*i.e.* the beam is below the diffraction limit for the on-axis radiation ω = *H*ω_0_. Therefore, the conditions of applying equation (30) are 

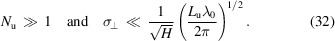

If the second condition is not satisfied, the more accurate result equation (26) should be referred.

**Figure 4:**
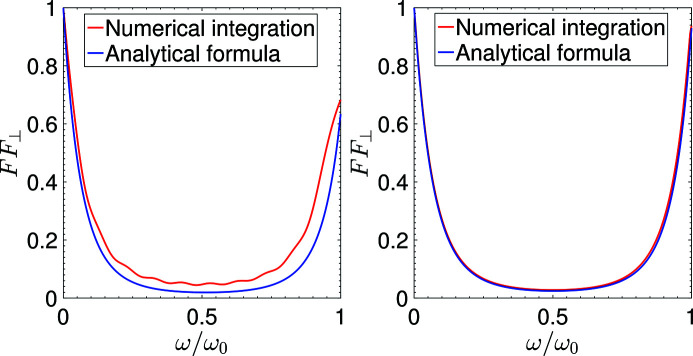
Comparison of the transverse form factor between that calculated from our simplified analytical formula equation (30) and that from the direct numerical integration of equation (12) for the case of *H* = 1, with *N*_u_ = 10 (left) and *N*_u_ = 100 (right). Other related parameters used in the calculation are: *E*_0_ = 400 MeV, λ_0_ = 13.5 nm, λ_u_ = 1 cm, *K* = 1.14, 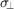 = 5 µm.

As a benchmark of our derivation, here we conduct some calculations of the transverse form factor based on direct numerical integration of equation (12), and compare them with our simplified analytical formula equation (30). The parameters used are for the envisioned EUV SSMB to be presented in Section 6. As can be seen in Fig. 4 ▸, their agreement when *N*_u_ = 100 is remarkably good. Even in the case of *N*_u_ = 10, the agreement is still satisfactory. There are two reasons why the agreement is better in the case of a large *N*_u_. The first is that in the derivation we have made use of the sharpness of *F*(ε), whose width is 1/*N*_u_. The second is that *S* ∝ 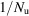 with a given transverse beam size and undulator period length, and our simplified analytical formula equation (30) applies when *S*(ω = *H*ω_0_) 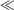 1.

**Figure 5:**
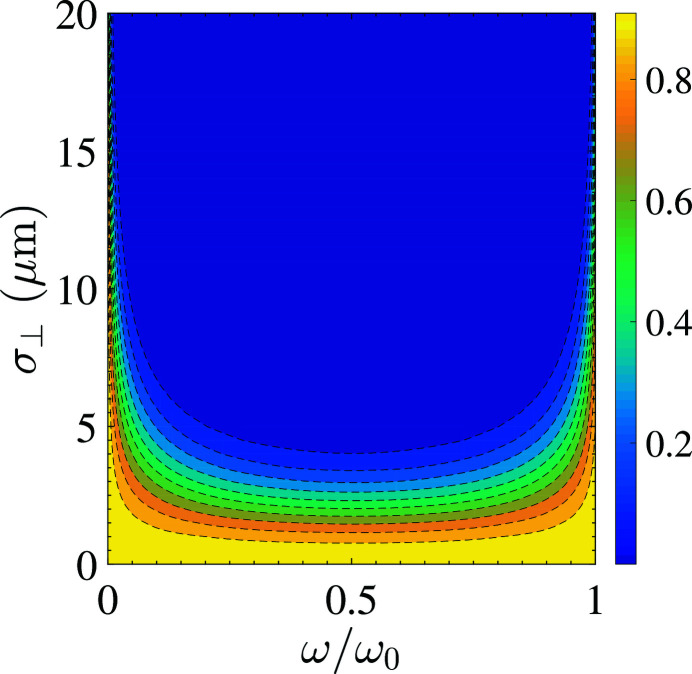
Flat contour plot of the transverse form factor 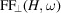 for *H* = 1, as a function of the radiation frequency ω and transverse electron beam size 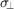 , calculated using our simplified analytical formula equation (30). Parameters used in the calculation are: *E*_0_ = 400 MeV, λ_0_ = 13.5 nm, λ_u_ = 1 cm, *K* = 1.14, *N*_u_ = 79.

To appreciate the implication of the generalized transverse form factor further, an example flat contour plot of the transverse form factor as a function of the radiation frequency ω and transverse electron beam size 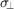 is shown in Fig. 5 ▸. As can be seen, a large transverse electron beam size will suppress the off-axis red-shifted coherent radiation due to the projected bunch lengthening from the transverse size, and thus the effective bunching factor degradation, when observed off-axis. Therefore, a large transverse electron beam size will make the coherent radiation more collimated in the forward direction, and more narrow-banded around the harmonic lines. Note, however, that not only the red-shifted radiation is suppressed – the radiation strength of each harmonic line ω = *H*ω_0_ actually also decreases with the increase of transverse electron beam size, the reason for which we have just explained.

Now we evaluate the bandwidth and opening angle of the radiation at different harmonics due to the transverse form factor. In particular, we are interested in the case where the off-axis red-shifted radiation is significantly suppressed by the transverse size of the electron beam, which requires that 

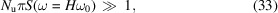


*i.e.*


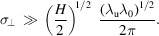

Note that to apply equation (30) we still need the conditions in equation (32). For example, to apply the analytical estimation for the example EUV SSMB calculation to be presented in Section 6, in which λ_u_ = 1 cm, λ_0_ = 13.5 nm and *N*_u_ = 79, we need 1.3 µm 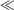 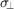 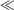 41 µm. The typical transverse electron beam size in an EUV SSMB ring is in this range.

With these conditions satisfied, the value of the exponential factor in equation (30) is more sensitive to the change of ω, compared with the universal function 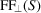 . Therefore, here we consider only the exponential term when ω is close to *H*ω_0_. We want to know the value of ω at which the exponential term gives

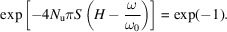

Putting in the definition of

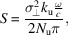

we have

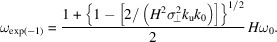

Then 

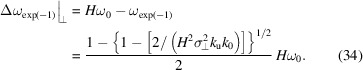

As

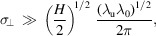

then

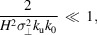

we have 

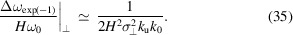

Correspondingly, the opening angle of the coherent radiation due to the transverse form factor is 

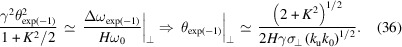

It is interesting to note that 

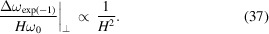

As a comparison, the relative bandwidth at the harmonics due to the longitudinal form factor is 

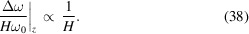

Note also that 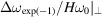 and 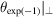 are independent of *N*_u_, although the approximations adopted in the derivation actually involve conditions related to *N*_u_.

## 4. Radiation power and spectral flux at harmonics

In many cases, the microbunching is formed based on an electron bunch much longer than the radiation wavelength, for example in an FEL or coherent harmonic generation (CHG). In these cases, the linewidths of the longitudinal form factor at the harmonics are usually even narrower than that given by the transverse form factor. This also means that the coherent radiation of a long continuous electron bunch based microbunching will be dominantly in the forward direction, as the bunching factor of the off-axis red-shifted frequency is suppressed very quickly compared with the on-axis resonant ones. For a more practical application, here we derive the coherent radiation power and spectral flux at the undulator radiation harmonics in these cases. As we will see, the results can be viewed as useful references for SSMB radiation.

We assume that the long electron bunch, before microbunching, is Gaussain. Here we assume that the transverse form factors around the harmonics do not change much, *i.e.* we assume 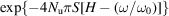 ≃ 1 when ω is close to *H*ω_0_. Therefore, we only need to take into account the Gaussian shape of the longitudinal form factors at the harmonics. The bandwidth of the longitudinal form factor for a Gaussian bunch of length σ_*z*_ is Δω|_*z*_ = 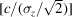 . Therefore, the coherent radiation energy at the *H*th harmonic is 

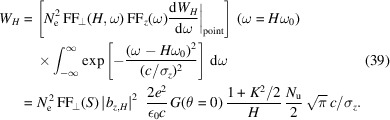

For a planar undulator, the σ-mode radiation dominates and from equation (16) we have 

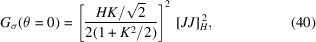

in which the denotation

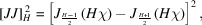

with χ = 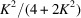 , is used. Note, however, that the above expression is meaningful only for an odd *H*, as the on-axis even harmonic radiation is rather weak. The peak power of the odd-*H*th harmonic coherent radiation is then 

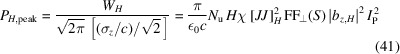

where *I*_P_ = 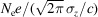 is the peak current of the Gaussian bunch before microbunching. For a more practical application of the derived formula, we put in the numerical value of the constants, and arrive at 

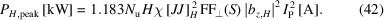

Note that the above formulas apply when the radiation slippage length *N*_u_λ_0_ is smaller than the bunch length σ_*z*_. If not, the above formulas will overestimate the coherent radiation peak power, as the r.m.s. radiation pulse length is then longer than 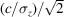 . Note also that, given the same bunch charge and form factors, *P*_*H*, peak_ ∝ 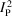 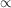 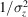 and *W*_*H*_ ∝ 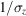 . The reason why a shorter bunch radiates more total energy is because more particles are within the coherence length.

At a first glance of equation (41), the coherent radiation power *P*_coh_ seems to be proportional to *N*_u_, while an intuitive picture of the longitudinal coherence length *l*_coh_ ∝ *N*_u_ says that the scaling should be *P*_coh_ ∝ 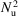 , as the electron number within the coherence length is proportional to *N*_u_. This is actually because 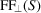 is also a function of *N*_u_. It is interesting to note that 

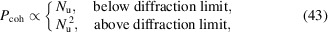

which can be obtained from the asymptotic expressions of 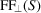 as shown in equation (29). So for a given transverse beam size, 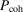 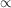 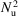 at first when *N*_u_ is small. When *N*_u_ is large enough such that the electron beam is below the diffraction limit, then *P*_coh_ ∝ *N*_u_. Physically this is because, with the increase of *N*_u_, the diffraction of the radiation means that radiation from one particle cannot effectively affect the particles far in front of it, as the on-axis field from this particle becomes weaker with the increase of the radiation slippage length.

Our derivation of the coherent radiation power above is for a Gaussian bunch-based microbunching. For a coasting or DC beam, we just need to replace *I*_P_ in equation (41) by the average current *I*_A_, and the peak power is then the average power. For a helical undulator, we need to replace 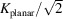 with *K*_helical_, and 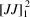 with 1, in the evaluation of the radiation power at the fundamental frequency.

We remind the reader that equation (41) is for the case of a long continuous bunch-based microbunching, for example in FELs and CHG. In SSMB, the microbunches are cleanly separated from each other according to the modulation laser wavelength, and usually the radiation wavelength is at a high harmonic of the modulation laser. Therefore, there could actually be significant red-shifted radiation generated in SSMB as we will see in the example calculation in Section 6. If we put the average current of SSMB into equation (41), it evaluates the radiation power whose frequency content is close to the on-axis harmonic and will underestimate the overall radiation power. We will see this argument clearer in Section 6.

After investigating the radiation power, let us now have a look at the spectral flux, which is the number of photons per unit time in a given small bandwidth. The spectral flux of coherent radiation at the odd-*H*th harmonic can be calculated according to 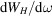 as 

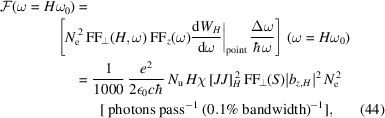

where ℏ is the reduced Planck’s constant. Again we put in the numerical value of the constants, and arrive at 

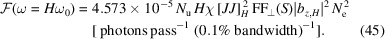

Note that the above spectral flux is for a single pass of the microbunched electron beam through the radiator undulator. For the evaluation of the average spectral flux in an SSMB storage ring, we need to multiply it by the number of microbunches passing a fixed location in one second, namely 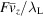 , with *F* being the filling factor of microbunches in the ring, 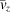 being the average longitudinal speed of an electron wiggling in the modulator undulator, and λ_L_ being the modulation laser wavelength. We remind the reader that the above statement means we do not account for the radiation overlapping between different microbunches if the radiation slippage length is larger than 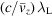 . If there is such radiation overlapping, the flux will be boosted further since the electrons in neighboring microbunches can now cohere with each other. To give the reader a more concrete feeling about the high spectral flux in SSMB, we just need to multiply the spectral flux of the usual incoherent undulator radiation by a factor of 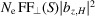 , with *N*_e_ being the number of electrons per microbunch. For example, in the envisioned EUV SSMB to be presented in Section 6, *N*_e_ = 2.2 × 10^4^, and 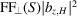 can be as large as 0.1. Therefore, the EUV spectral flux in an SSMB storage ring can thus be three orders of magnitude higher than that in a conventional synchrotron source.

## 5. Statistical properties of radiation

In the previous sections, we have ignored the quantum discrete nature of radiation. In addition, we have derived the coherent radiation properties using a smooth distributed charge, *i.e.* we have treated the charge as a continuum fluid. The number of photons radiated from a charged particle beam actually fluctuates from turn to turn or bunch to bunch if the quantum nature of the radiation and the pointlike nature of the electrons are taken into account (Goodman, 2015 ▸). The first mechanism exists even if there is only one electron, and the second mechanism is related to the interference of fields radiated by different electrons (Lobach *et al.*, 2020 ▸). Using classical language, the second fluctuation mechanism is from the fluctuation of the bunching factor or form factor of the electron beam.

There have been studies on the statistical properties of the radiation in FELs (Saldin *et al.*, 1998 ▸) and also storage-ring-based synchrotron radiation sources, for example the recent seminal work of Lobach *et al.* (2020 ▸, 2021*a* ▸,*b* ▸). Rich information about the electron beam is embedded in the radiation fluctuations, or more generally the statistical properties of the radiation. For example, the turn-by-turn fluctuation of the incoherent undulator radiation can be used to measure the transverse emittance of the electron beam (Lobach *et al.*, 2021*b* ▸). The previous treatment, however, has usually been for cases where the bunch length is much longer than the radiation wavelength, *i.e.* the radiation is temporally incoherent (in SASE FEL, incoherent at the beginning). In SSMB, the bunch length is comparable with or shorter than the desired radiation wavelength, and the dominant radiation is temporally coherent. Although numerical calculation is possible following the general theoretical formulation, an analytical formula for the fluctuation in this temporally coherent radiation dominant regime is still of value for a better understanding of the physics and investigation of its potential applications.

### 5.1. Pointlike nature of electrons

Here for SSMB we consider first the second mechanism of fluctuation, *i.e.* the radiation power fluctuation arising from the pointlike nature of the radiating electrons. In this section, to simplify writing, we use the vector notation 

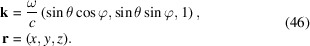

Then the bunching factor with the pointlike nature of electrons taken into account is 

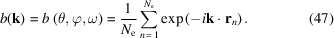

First we want to evaluate the coherent radiation power fluctuation at a specific frequency and observation angle. As the radiation power is proportional to 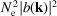 , we therefore need to know the fluctuation of |*b*(**k**)|^2^. Since 

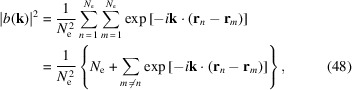

we have 

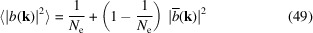

with 

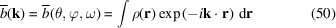

being the bunching factor we calculated before using a continuum fluid charge distribution in equation (3). As can be seen from equation (49), when *N*_e_ = 1, which corresponds to the case of a single point charge, then 〈|*b*(**k**)|^2^〉 = 1. When *N*_e_ ≫ 1 and 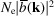 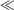 1, which corresponds to the case of incoherent radiation dominance, then 〈|*b*(**k**)|^2^〉 = 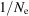 . When *N*_e_ ≫ 1 and 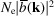 ≫ 1, which corresponds to the case of coherent radiation dominance, then 〈|*b*(**k**)|^2^〉 = 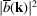 . These results are as expected.

The calculation of 〈|*b*(**k_1_**)|^2^|*b*(**k_2_**)|^2^〉 is more involved. More specifically, 

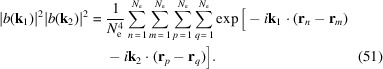

The 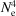 terms in this summation can be placed in 15 different cases, as shown in Table 1 ▸. Corresponding to the 15 cases, we have 

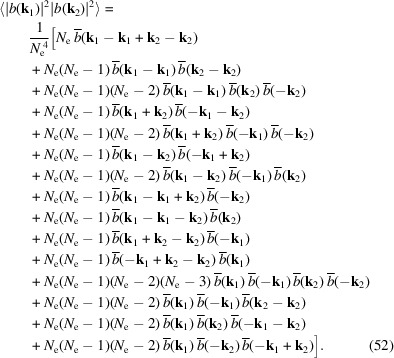

The above results can be re-organized as 

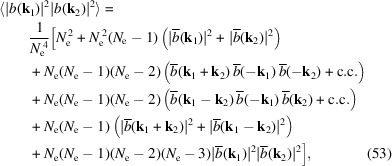

in which c.c. means the complex conjugate.

If **k**_1_ = **k**_2_ = **k**, then 

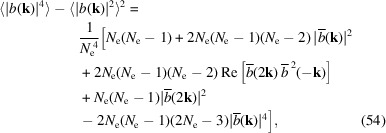

where Re[...] means taking the real part of a complex number.

When *N*_e_ ≫ 1 and 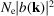 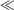 1, which is the case for incoherent radiation dominance, we have 〈|*b*(**k**)|^2^〉 = 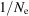 and 

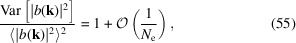

where Var[...] means the variance. Therefore, the relative fluctuation of incoherent radiation is relatively large. This is also the reason why SASE-FEL radiation has a large shot-to-shot power fluctuation. When *N*_e_ ≫ 1 and *N*_e_|*b*(**k**)|^2^ ≫ 1, which corresponds to the case of coherent radiation dominance like that in SSMB, we have 

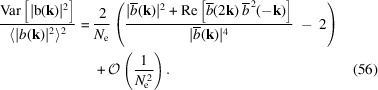

The above equation is the main result of our analysis of the bunching factor fluctuation for the regime of coherent radiation dominance, and to our knowledge is new. As mentioned, the radiation power at frequency ω is proportional to 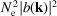 ; the formula can thus be used to evaluate the coherent radiation power fluctuation at a specific frequency and observation angle. If the transverse electron beam size is zero, or if we observe on-axis, then we can just replace *b*(**k**) with *b*_*z*_(ω) in the above formula.

**Figure 6:**
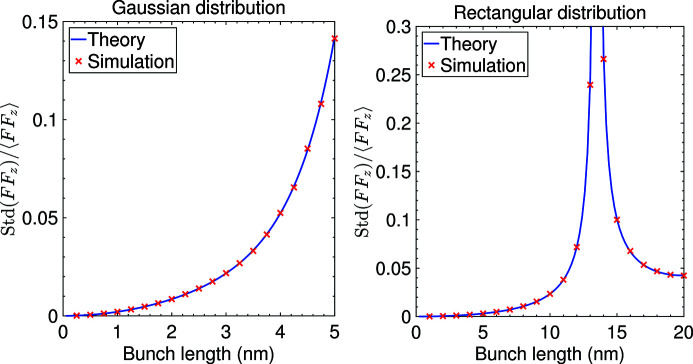
Fluctuation of the longitudinal form factor at 13.5 nm versus bunch length with *N*_e_ = 2.2 × 10^4^. The bunch distribution is assumed to be Gaussian in the left image and rectangular in the right image, and the theoretical fluctuation is calculated according to equation (56), omitting the term 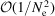 . For each parameters choice, 1 × 10^4^ simulations have been conducted to obtain the fluctuation.

Now we conduct some numerical simulations to confirm our analysis of coherent radiation fluctuation. As can be seen from Fig. 6 ▸, which corresponds to the cases of a Gaussian and a rectangular distributed bunch, the simulation results agree well with our theoretical prediction.

After investigating the expectation and variance of |*b*(**k**)|^2^, one may be curious about its more detailed distribution. It can be shown that when *N*_e_|*b*(**k**)|^2^ ≫ 1 the distribution |*b*(**k**)|^2^ tends asymptotically toward Gaussian.

As explained before, for a fixed frequency ω, there is a range of polar angles θ which can contribute. To evaluate the overall radiation power fluctuation at a specific frequency ω, we then need to know the fluctuation of the form factor FF(ω) which involves calculation depending on the specific radiation process. For undulator radiation, it appears to be not so easy to obtain a concise closed-form analytical formula to evaluate the total radiation power fluctuation when the beam has a finite transverse beam size. So here we refer to numerical calculation to give the reader a more concrete feeling about the impact of transverse size on coherent radiation power fluctuation.

**Figure 7:**
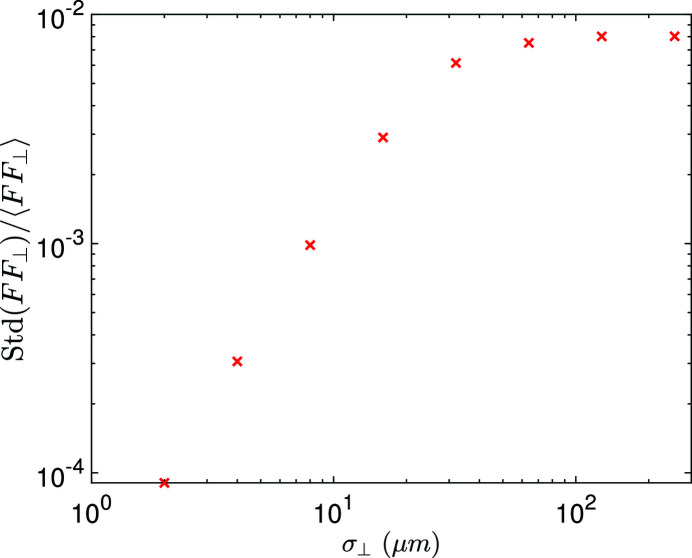
Fluctuation of the transverse form factor at 13.5 nm versus transverse beam size with *N*_e_ = 2.2 × 10^4^. The bunch is assumed to have zero length and is a round Gaussian in the transverse plane. For each parameter choice, 1 × 10^3^ simulations have been conducted to obtain the fluctuation.

For simplicity, we assume that the bunch length is zero and focus on the fluctuation of transverse form factor. As can be seen from the simulation result in Fig. 7 ▸, the larger the transverse beam size, the larger the transverse form factor fluctuation. We also notice that in a typical parameter set of the envisioned EUV SSMB the fluctuation of 13.5 nm radiation power due to the finite transverse size is small. For example, if 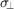 = 16 µm, then the relative fluctuation of the transverse form factor as shown in Fig. 7 ▸ is 0.3%; whereas the relative fluctuation of the longitudinal form factor at 13.5 nm when σ_*z*_ = 3 nm according to equation (56) is about 2%. Assuming that the beam is transverse–longitudinal decoupled, then 

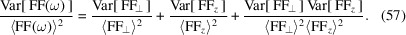

Therefore, for the envisioned EUV SSMB, the fluctuation of the longitudinal form factor dominates.

After investigating the power fluctuation at a specific frequency ω, now we look into the radiation energy fluctuation gathered within a finite frequency bandwidth and a finite angle acceptance. We use a filter function FT(θ, φ, ω) to account for the general case of frequency filter, angle acceptance and detector efficiency. The expectation of the gathered photon energy and photon energy squared are 

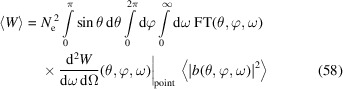

and 

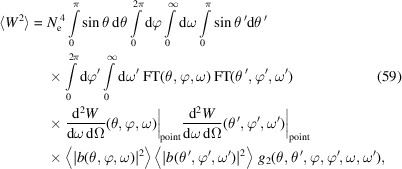

where 

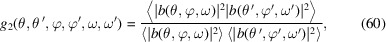

whose calculation can follow a similar approach of calculating 〈|*b*(**k**_1_)|^2^|*b*(**k**_2_)|^2^〉 in equation (52). The relative fluctuation of the gathered photon energy is 

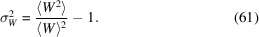




### 5.2. Quantum nature of radiation

As mentioned, there is another source of fluctuation, *i.e.* the quantum discrete nature of radiation. As a result of Campbell’s theorem (Campbell, 1909 ▸), we know that for Poisson photon statistics the variance of the photon number arising from this equals its expectation value. With both contributions from the pointlike nature of electrons and the quantum nature of radiation taken into account, the relative fluctuation of the radiation power or energy at a given frequency and a specific observation angle is 

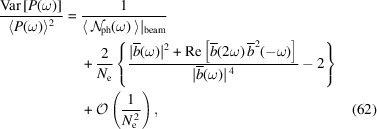

in which 

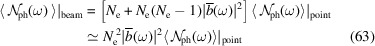

is the expected radiated photon number from the electron beam, and 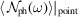 is the expected radiated photon number from a single electron. Note that to obtain a non-zero expected photon number 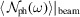 , a finite frequency bandwidth is needed. Therefore, equation (62) actually applies to a finite frequency bandwidth close to ω where 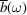 does not change much.

From equation (62), it is interesting to note that with the narrowing of the energy bandwidth acceptance, *i.e.* the decrease of 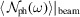 , the contribution to the relative fluctuation from the quantum nature of radiation increases, while the contribution from the pointlike nature of the electron does not change. This reflects the fact that one fluctuation is quantum, while the other is classical.

Note that in our case of interest, SSMB, 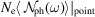 is usually much larger than 1, so the second term in equation (62) dominates. In other words, the fluctuation due to the pointlike nature of electrons dominates. Only when 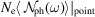 is close to 1 will the first term become significant compared with the second term.

### 5.3. Potential applications

As the statistical property of the radiation embeds rich information about the electron beam, the innovative beam diagnostics method can be envisioned by making use of this fact. Here we propose an experiment to measure the sub-ps bunch length accurately at a quasi-isochronous storage ring, for example the MLS, at a low beam current, by measuring and analyzing the fluctuation of the coherent THz radiation generated from the electron bunch. Equation (62) or some numerical code based on the analysis presented in this section will be the theoretical basis for the experimental proposal. In principle, we can also deduce the beam transverse distribution by measuring the two-dimensional distribution of the radiation fluctuation. More novel beam diagnostics methods may be invented for SSMB and future light sources by making use of the statistical properties of radiation. One advantage of using radiation fluctuation in diagnostics is that it has a less stringent requirement on the calibration of detectors.

## 6. Example calculation for envisioned EUV SSMB

To summarize our investigations on the average and statistical properties of SSMB radiation, here we present an example calculation for the envisioned EUV SSMB. In the envisioned SSMB-based EUV light source, the microbunch length is σ_*z*_ ≃ 3 nm at the radiator where 13.5 nm coherent EUV radiation is generated, and the bunches in this 3 nm microbunch train are separated from each other by a distance λ_L_ = 1064 nm = 79 × 13.5 nm, which is the modulation laser wavelength. The radiator is assumed to be an undulator. The beam at the radiator can be round or flat depending on the lattice scheme, and its transverse size can range from a couple of micrometres to a couple of tens of micrometres. As our goal is to give the reader a picture of the radiation characteristics, here for simplicity we consider the case of a round beam. We remind the reader that the parameters used in this example of a EUV SSMB radiation calculation are for illustration and are not optimized.

### 6.1. Average property

**Figure 8:**
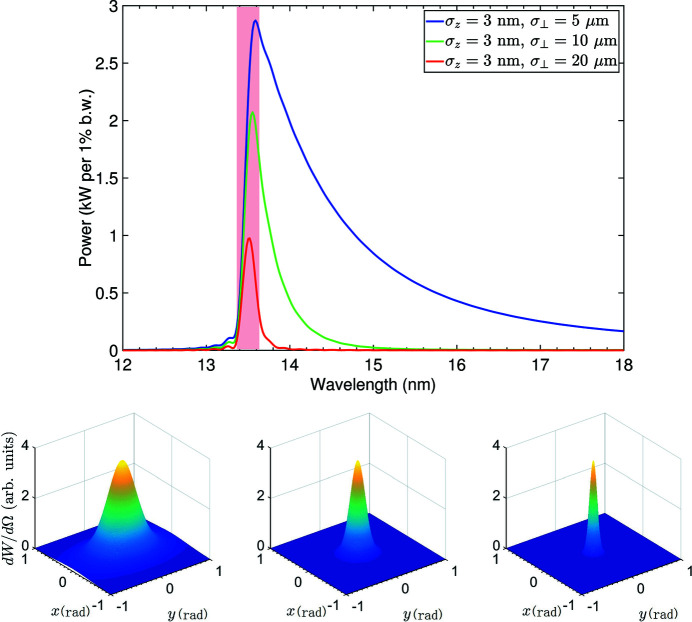
An example EUV SSMB radiation calculation with a microbunch length of σ_*z*_ = 3 nm and different transverse sizes 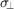 . The top image shows the energy spectrum. Corresponding to 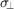 = 5, 10 and 20 µm, the total radiation power is 39 kW, 7 kW and 1.7 kW, respectively. The shaded area corresponds to a wavelength of 13.5 ± 0.135 nm. The lower images show the spatial distribution of the radiation energy plotted in a 3D view in the (*x*, *y*) space with *x* = 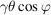 , *y* = 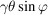 . From left to right: 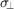 = 5, 10 and 20 µm. Parameters used for the calculation are: *E*_0_ = 400 MeV, *I*_avg_ = 1 A, λ_L_ = 1064 nm, λ_r_ = 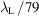 = 13.5 nm, λ_u_ = 1 cm, *K* = 1.14, *N*_u_ = 79.

First we present the results for the average properties of the EUV radiation. The calculation is based on equations (7), (11) and (16), and the result is shown in Fig. 8 ▸. The upper part of the figure shows the radiation energy spectrum. The lower part shows the spatial distributions of the radiation energy. The total radiation power is calculated according to 

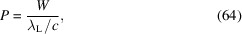

where *W* is the total radiation energy loss of each microbunch. For the example radiator undulator parameters, corresponding to 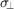 = 5, 10 and 20 µm, the total radiation power is 39 kW, 7 kW and 1.7 kW, respectively. As a reference, the radiation power calculated based on equation (41) for these three transverse beam sizes is 1.8 kW, 1.5 kW and 0.93 kW, respectively. The reason why equation (41) gives a smaller value than the overall power as explained is that it does not take into account the red-shifted part of the radiation. Therefore, equation (41) can be used to evaluate the lower bound of the radiation power from SSMB, once the parameter set of the electron beam and radiator undulator is given. It can be seen that, generally, 1 kW EUV radiation power can be straightforwardly anticipated from a 3 nm microbunch train with an average beam current of 1 A. Note that, for simplicity, in this example calculation the filling factor of microbunches in the ring is assumed to be 100%, *i.e.* one microbunch per modulation laser wavelength. Then 1 A average current corresponds to the number of electrons per microbunch *N*_e_ = 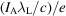 = 2.2 × 10^4^, if the modulation laser wavelength is λ_L_ = 1064 nm.

Another important observation is that the spectral and spatial distribution of SSMB radiation depends strongly on the transverse size of the electron beam. A large transverse size results in a decrease of the overall radiation power, and will also make the radiation more narrow-banded and collimated in the forward direction. This is an important observation drawn from our investigation on the generalized transverse form factor. Using the example parameters, *i.e.* *E*_0_ = 400 MeV, λ_L_ = 1064 nm, λ_0_ = 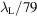 = 13.5 nm, λ_u_ = 1 cm, *N*_u_ = 79 and *K* = 1.14, if 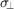 = 10 µm, then according to equations (35) and (36) the relative bandwidth and opening angle due to the transverse form factor can be calculated to be 

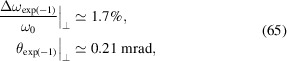

which is in agreement with the result presented in Fig. 8 ▸.

The energy spectrum and spatial distributions presented in Fig. 8 ▸ are for that of a single microbunch. For the energy spectrum of radiation from a periodic microbunch train, such as that in SSMB, we just need to multiply it by a periodic delta function in the frequency domain whose frequency separation is the modulation laser frequency, the situation of which is similar to that of conventional synchrotron radiation in a storage ring. Corresponding to these delta function lines in the energy spectrum, there will be ring-shaped peaks in the spatial distribution of the coherent radiation as a result of the interference of radiation from different microbunches. The polar angles of these rings, corresponding to the delta function lines in the energy spectrum, are determined by the off-axis resonant condition. Note, however, that the electron beam energy spread and angular divergence will make the linewidth of these delta function lines become non-zero. For example, the relative bandwidth of the radiation caused by an energy spread of σ_δ_ is 2σ_δ_.

As a result of the high-power and narrow-band feature of the SSMB radiation, a high EUV photon flux of 6 × 10^15^ photons s^−1^ within a 0.1 meV bandwidth can be obtained, if we can realize an EUV power of 1 kW per 1% bandwidth as shown in Fig. 8 ▸. We remind the reader that the radiation waveform of SSMB is actually a CW or quasi-CW one, if an induction linac is used as the energy compensation system and the microbunches occupy the ring with a large filling factor, as assumed in the example calculation. This kind of CW or quasi-CW narrow-band light is favored in ARPES to minimize the space-charge-induced energy shift, spectral broadening and distortion of photoelectrons in a pulsed photon source based ARPES (Zhou *et al.*, 2005 ▸; Hellmann *et al.*, 2009 ▸; Tamai *et al.*, 2013 ▸). Therefore, the high photon flux within a narrow bandwith, together with its CW or quasi-CW waveform, make SSMB a promising light source for ultrahigh-resolution ARPES. Such a powerful tool may have profound impact on fundamental physics research, for example to probe the energy gap distribution and electronic states of superconducting materials like magic angle graphene (Cao *et al.*, 2018 ▸).

### 6.2. Statistical properties

Now we present the result for the statistical properties of the radiation. For the case of a Gaussian bunch with σ_*z*_ = 3 nm and *N*_e_ = 2.2 × 10^4^, from equation (56) we know that the relative fluctuation of the turn-by-turn or microbunch-by-microbunch on-axis 13.5 nm coherent radiation power will be around 2%.

**Figure 9:**
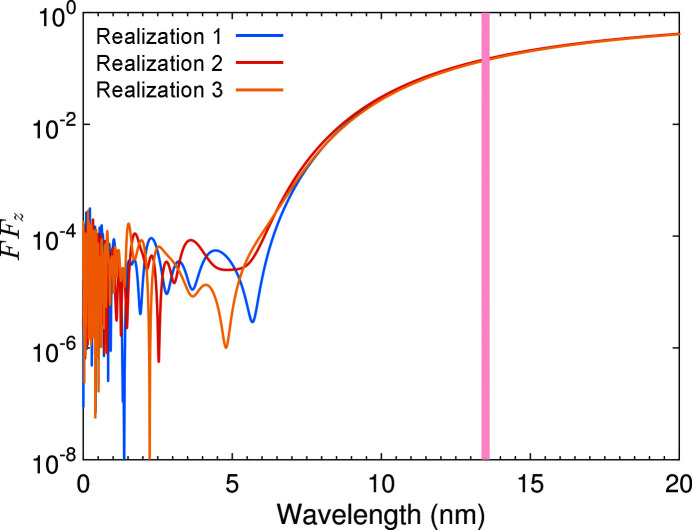
Spectra of the longitudinal form factor of three possible realizations of a Gaussian microbunch length of σ_*z*_ = 3 nm and *N*_e_ = 2.2 × 10^4^. The shaded area corresponds to a wavelength of 13.5 ± 0.135 nm.

Figure 9 ▸ gives an example plot for the longitudinal form factor spectrum of three possible realizations of such a Gaussian microbunch. As can be seen, the spectrum is noisy mainly in the high-frequency or short-wavelength range. Our EUV radiation is mainly at a wavelength close to 13.5 nm, and the longitudinal form factor close to this frequency fluctuates together from turn to turn, or bunch to bunch. As shown in Fig. 7 ▸ and discussed before, for the envisioned EUV SSMB the transverse form factor fluctuation is much smaller than that of the longitudinal form factor. So the overall radiation power fluctuation is also about 2% as analyzed above. This fluctuation is also the microbunch center of motion fluctuation induced by the coherent radiation. Although it is a small fluctuation, its beam dynamics effects need further study.

Note that this 2% fluctuation of radiation power should have negligible impact for the application in EUV lithography, since the revolution frequency of the microbunch in the ring is rather high (MHz), let alone if we consider that there is actually a microbunch for each modulation laser wavelength and the radiation waveform is CW or quasi-CW.

### 6.3. Discussions

To resolve possible concerns on the validity of the short bunch length and high average current used in the example calculation, here we present a short discussion on the related single-particle and collective effects in SSMB. We recognize that realizing a steady-state bunch length as short as the nanometre level in an electron storage ring is non-trivial. Both global and local momentum compaction should be minimized to confine the longitudinal beta function, and therefore the longitudinal emittance, in an electron storage ring (Deng *et al.*, 2020*a* ▸, 2021*c* ▸; Zhang *et al.*, 2021 ▸). By invoking this principle in the lattice design, a bunch length as short as tens of nanometres can be realized in a storage ring (Pan, 2020 ▸), with a momentum compaction factor of 1 × 10^−6^ and modulation laser power of 1 MW. An intra-cavity power of 1 MW is the state-of-art level of present optical enhancement cavity technology. Therefore, to realize nanometre bunch length at the radiator, we need to compress the bunch further. There are two scenarios being actively studied by the Tsinghua SSMB task force (Tang & Deng, 2022 ▸), namely the longitudinal strong focusing scheme and the transverse–longitudinal coupling scheme. The longitudinal strong focusing scheme is similar to its transverse counterpart which is the basis of modern particle accelerators. In such a scheme, the longitudinal beta function and therefore the bunch length is strongly focused at the radiator, and the synchrotron tune of the beam in the ring can be at the level of 1 (Zhang, 2022 ▸). Although nanometre bunch length can be realized, this scheme requires a large modulation laser power (100 MW level), thus causing the optical cavity to work only in the pulsed laser mode, and the average output radiation power is thus limited. To lower the modulation laser power, the transverse–longitudinal coupling scheme is thus applied in a clever way by taking advantage of the fact that the vertical emittance in a planar storage ring is rather small (Feng & Zhao, 2017 ▸; Deng *et al.*, 2021*b* ▸; Deng, 2022 ▸). We refer to this turn-by-turn transverse–longitudinal coupling-based bunch compression scheme as the generalized longitudinal strong focusing, in which the phase space manipulation is 4D or 6D, in contrast to the conventional longitudinal strong focusing where the phase space manipulation is 2D. This generalized longitudinal strong focusing scheme can relax the modulation laser power, but its nonlinear dynamics optimization is a challenging task which we are trying to tackle.

Concerning the high average current, there are two collective effects of special importance, namely the intra-beam scattering (IBS) and coherent synchrotron radiation (CSR). IBS will affect the equilibrium emittance and thus can have an impact on the radiation power and also the modulation laser power in the generalized longitudinal strong focusing scheme. The IBS effect in an SSMB ring thus needs careful optimization and the operation beam energy is also mainly determined by IBS. CSR is the reason why SSMB can provide powerful radiation. On the other hand, CSR is also the effect which sets the upper limit of the stable beam current. Chao *et al.* (2016 ▸) showed some preliminary evaluation of the stable beam current for SSMB based on the 1D model of CSR-driven microwave instability. The investigation in this paper implies that the transverse dimension of the electron beam can have a large impact on the coherent radiation in SSMB. In addition, the bunch lengthening from the transverse emittance in an SSMB storage ring can easily dominate the bunch length at many dispersive places of the ring, as the transverse size of microbunches is much larger than its longitudinal length. This bunch lengthening might be helpful in mitigating unwanted CSR. The 3D effect of the coherent radiation is expected to be also helpful in improving the stable beam current. With these beneficial arguments in mind, we realize that CSR in SSMB still deserves special attention. For example, the coherent radiation in the laser modulator could potentially also drive single-pass and multi-pass collective instabilities in an SSMB storage ring (Tsai *et al.*, 2021 ▸; Tsai, 2022*a* ▸,*b* ▸). A more in-depth study of collective effects in SSMB is ongoing.

For completeness of the discussion, here we also elaborate on the conditions for applying the rigid beam approximation in evaluating the SSMB radiation. For the example EUV SSMB parameters used in the calculation, *L*_u_ = 0.79 m and *R*_56,r_ = 2*N*_u_λ_r_ = 2.128 µm. The rigid beam approximation applies when β_*x*,*y*_ > *L*_u_ and β_*z*_ > *R*_56,u_. Assuming that the beam distribution in the transverse and longitudinal phase spaces are upright in the middle of the radiator, then we require that β_*x*,*y*_ > 0.79 m and σ_δ_ < 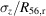 ≃ 1.5 × 10^−3^ to apply the rigid beam approximation. Such requirements should not be difficult to meet. Further, if we want to use the derived simplified transverse form factor equation (30), according to equation (32), then we need 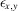 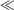 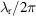 = 2 nm, which is also nominally satisfied in an EUV SSMB storage ring.

## 7. Summary

A theoretical and numerical study of the average and statistical properties of coherent radiation from an SSMB storage ring is presented in this paper. For the theoretical investigations, equations (30), (41) and (56) are the main results. Our work shows that average-power 1 kW EUV radiation can be obtained from an SSMB light source, provided that an average current of 1 A and a microbunch train with bunch length of 3 nm can be formed at the radiator which is assumed to be an undulator. Such a high-power EUV source is highly desired by the semiconductor industry for lithography. Together with its narrow-band feature and quasi-CW waveform, SSMB radiation is promising in realizing an ultrahigh-resolution ARPES, which holds profound impact on condensed matter physics studies. We have shown that the narrow-band feature of SSMB radiation is strongly correlated with the finite transverse electron beam size, an observation drawn from our investigation of the generalized transverse form factor. Some important results concerning the statistical properties of SSMB radiation are also presented, with a brief discussion on its potential application, for example the beam diagnostics. The presented work is of value for the development of SSMB and to better serve the synchrotron radiation user community. The next step is to further investigate the impact of the electron beam distribution in 6D phase space on the radiation, and the radiation acting back on the electron beam.

## Figures and Tables

**Table 1:** The 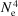 terms in the quadruple sum of equation (51) can be placed in 15 different classes, as shown by Goodman (2015 ▸)

Item number	Index relations	Number of terms
(1)	*n* = *m* = *p* = *q*	*N*_e_
(2)	*n* = *m*, *p* = *q*, *n* ≠ *p*	*N*_e_(*N*_e_ − 1)
(3)	*n* = *m*, *p* ≠ *q* ≠ *n*	*N*_e_(*N*_e_ − 1)(*N*_e_ − 2)
(4)	*n* = *p*, *m* = *q*, *n* ≠ *m*	*N*_e_(*N*_e_ − 1)
(5)	*n* = *p*, *m* ≠ *q* ≠ *n*	*N*_e_(*N*_e_ − 1)(*N*_e_ − 2)
(6)	*n* = *q*, *m* = *p*, *n* ≠ *m*	*N*_e_(*N*_e_ − 1)
(7)	*n* = *q*, *m* ≠ *p* ≠ *n*	*N*_e_(*N*_e_ − 1)(*N*_e_ − 2)
(8)	*n* = *m* = *p*, *n* ≠ *q*	*N*_e_(*N*_e_ − 1)
(9)	*n* = *m* = *q*, *n* ≠ *p*	*N*_e_(*N*_e_ − 1)
(10)	*n* = *p* = *q*, *n* ≠ *m*	*N*_e_(*N*_e_ − 1)
(11)	*p* = *q* = *m*, *n* ≠ *m*	*N*_e_(*N*_e_ − 1)
(12)	*n* ≠ *m* ≠ *p* ≠ *q*	*N*_e_(*N*_e_ − 1)(*N*_e_ − 2)(*N*_e_ − 3)
(13)	*p* = *q*, *n* ≠ *m* ≠ *p*	*N*_e_(*N*_e_ − 1)(*N*_e_ − 2)
(14)	*m* = *q*, *n* ≠ *m* ≠ *p*	*N*_e_(*N*_e_ − 1)(*N*_e_ − 2)
(15)	*m* = *p*, *n* ≠ *m* ≠ *q*	*N*_e_(*N*_e_ − 1)(*N*_e_ − 2)

## References

[bb1] Altarelli, M., Brinkmann, R., Chergui, M., Decking, W., Dobson, B., Düsterer, S., Grübel, G., Graeff, W., Graafsma, H., Hajdu, J., Marangos, J., Pflüger, J., Redlin, H., Riley, D., Robinson, I., Rossbach, J., Schwarz, A., Tiedtke, K., Tschentscher, T., Vartaniants, I., Wabnitz, H., Weise, H., Wichmann, R., Witte, K., Wolf, A., Wulff, M. & Mikhail, Y. (2007). *The European X-ray Free-Electron Laser*. Technical design report. DESY XFEL Project Group, Hamburg, Germany.

[bb2] Bakshi, V. (2018). *EUV Lithography*, 2nd ed. SPIE Press.

[bb3] Bonifacio, R., Pellegrini, C. & Narducci, L. (1984). *AIP Conf. Proc.* **118**, 236–259.

[bb4] Campbell, N. (1909). *Proc. Camb. Philos. Soc.* **15**, 117–136.

[bb5] Cao, Y., Fatemi, V., Fang, S., Watanabe, K., Taniguchi, T., Kaxiras, E. & Jarillo-Herrero, P. (2018). *Nature*, **556**, 43–50. 10.1038/nature26160 29512651

[bb6] Carr, G. L., Martin, M. C., McKinney, W. R., Jordan, K., Neil, G. R. & Williams, G. P. (2002). *Nature*, **420**, 153–156. 10.1038/nature01175 12432385

[bb7] Chao, A., Granados, E., Huang, X., Ratner, D. & Luo, H.-W. (2016). *In Proceedngs of the 7th International Particle Accelerator Conference (IPAC’16)*, 8–13 May 2006, Busan, Korea, pp. 1048–1053. TUXB01.

[bb8] Chao, A. W. (2020). *Lectures on Accelerator Physics.* Singapore: World Scientific.

[bb9] Chao, A. W. & Chou, W. (2011). *Reviews of Accelerator Science and Technology*, Vol. 3, *Accelerators as Photon Sources.* Singapore: World Scientific.

[bb10] Cole, B., Williams, J., King, B., Sherwin, M. & Stanley, C. (2001). *Nature*, **410**, 60–63. 10.1038/35065032 11242038

[bb11] Courant, E. D. & Snyder, H. S. (1958). *Ann. Phys.* **3**, 1–48.

[bb12] Damascelli, A., Hussain, Z. & Shen, Z.-X. (2003). *Rev. Mod. Phys.* **75**, 473–541.

[bb13] Deng, X. (2022). *Theoretical and experimental studies on steady-state microbunching.* PhD thesis, Tsinghua University, Beijing, China.

[bb14] Deng, X., Chao, A., Feikes, J., Hoehl, A., Huang, W., Klein, R., Kruschinski, A., Li, J., Matveenko, A., Petenev, Y., Ries, M., Tang, C. & Yan, L. (2021*a*). *Nature*, **590**, 576–579. 10.1038/s41586-021-03203-0 33627811

[bb15] Deng, X., Huang, W., Li, Z. & Tang, C. (2021*b*). *Nucl. Instrum. Methods Phys. Res. A*, **1019**, 165859.

[bb16] Deng, X. J., Chao, A. W., Feikes, J., Huang, W. H., Ries, M. & Tang, C. X. (2020*a*). *Phys. Rev. Accel. Beams*, **23**, 044002.

[bb17] Deng, X. J., Chao, A. W., Huang, W. H. & Tang, C. X. (2021*c*). *Phys. Rev. Accel. Beams*, **24**, 094001.

[bb18] Deng, X. J., Klein, R., Chao, A. W., Hoehl, A., Huang, W. H., Li, J., Lubeck, J., Petenev, Y., Ries, M., Seiler, I., Tang, C. X. & Feikes, J. (2020*b*). *Phys. Rev. Accel. Beams*, **23**, 044001.

[bb19] Elder, F. R., Gurewitsch, A. M., Langmuir, R. V. & Pollock, H. C. (1947). *Phys. Rev.* **71**, 829–830.

[bb20] Emma, P., Akre, R., Arthur, J., Bionta, R., Bostedt, C., Bozek, J., Brachmann, A., Bucksbaum, P., Coffee, R., Decker, F., Ding, Y., Dowell, D., Edstrom, S., Fisher, A., Frisch, J., Gilevich, S., Hastings, J., Hays, G., Hering, P., Huang, Z., Iverson, R., Loos, H., Messerschmidt, M., Miahnahri, A., Moeller, S., Nuhn, H., Pile, G., Ratner, D., Rzepiela, J., Schultz, D., Smith, T., Stefan, P., Tompkins, H., Turner, J., Welch, J., White, W., Wu, J., Yocky, G. & Galayda, J. (2010). *Nat. Photon.* **4**, 641–647.

[bb21] Eriksson, M., van der Veen, J. F. & Quitmann, C. (2014). *J. Synchrotron Rad.* **21**, 837–842. 10.1107/S1600577514019286 25177975

[bb22] Feng, C. & Zhao, Z. (2017). *Sci. Rep.* **7**, 4724. 10.1038/s41598-017-04962-5 PMC5498574 28680101

[bb23] Galayda, J. N. (2018). *The LCLS-II: a high power upgrade to the LCLS.* Technical Report. SLAC National Accelerator Laboratory, Menlo Park, CA, USA.

[bb24] Goodman, J. W. (2015). *Statistical Optics.* John Wiley & Sons.

[bb25] Gover, A., Ianconescu, R., Friedman, A., Emma, C., Sudar, N., Musumeci, P. & Pellegrini, C. (2019). *Rev. Mod. Phys.* **91**, 035003.

[bb26] Hellmann, S., Rossnagel, K., Marczynski-Bühlow, M. & Kipp, L. (2009). *Phys. Rev. B*, **79**, 035402.

[bb27] Hemsing, E., Stupakov, G., Xiang, D. & Zholents, A. (2014). *Rev. Mod. Phys.* **86**, 897–941.

[bb28] Huang, Z. & Kim, K.-J. (2007). *Phys. Rev. ST Accel. Beams*, **10**, 034801.

[bb29] Jackson, J. D. (1999). *Classical Electrodynamics*, 3rd ed. New York: Wiley.

[bb30] Jiao, Y., Ratner, D. F. & Chao, A. W. (2011). *Phys. Rev. ST Accel. Beams*, ** 14**, 110702.

[bb31] Khan, S. (2017). *Nucl. Instrum. Methods Phys. Res. A*, **865**, 95–98.

[bb32] Kim, K.-J., Shvyd’ko, Y. & Reiche, S. (2008). *Phys. Rev. Lett.* **100**, 244802. 10.1103/PhysRevLett.100.244802 18643591

[bb33] Kondratenko, A. & Saldin, E. (1980). *Part. Accel.* **10**, 207–216.

[bb34] Krausz, F. & Ivanov, M. (2009). *Rev. Mod. Phys.* **81**, 163–234.

[bb35] Li, C., Feng, C., Jiang, B. & Chao, A. (2019). *Proceedingsof the 10th International Particle Accelerator Conference (IPAC’19)*, 19–24 May 2019, Melbourne, Australia, pp. 1507–1509. TUPGW045.

[bb36] Lobach, I., Lebedev, V., Nagaitsev, S., Romanov, A., Stancari, G., Valishev, A., Halavanau, A., Huang, Z. & Kim, K.-J. (2020). *Phys. Rev. Accel. Beams*, **23**, 090703. 10.1103/PhysRevLett.126.134802 33861120

[bb37] Lobach, I., Nagaitsev, S., Lebedev, V., Romanov, A., Stancari, G., Valishev, A., Halavanau, A., Huang, Z. & Kim, K.-J. (2021*a*). *Phys. Rev. Accel. Beams*, **24**, 040701. 10.1103/PhysRevLett.126.134802 33861120

[bb38] Lobach, I., Nagaitsev, S., Lebedev, V., Romanov, A., Stancari, G., Valishev, A., Halavanau, A., Huang, Z. & Kim, K.-J. (2021*b*). *Phys. Rev. Lett.* **126**, 134802. 10.1103/PhysRevLett.126.134802 33861120

[bb39] Lu, Y., Wang, X., Deng, X., Feng, C. & Wang, D. (2022). *Results Phys.* **40**, 105849.

[bb40] Lv, B., Qian, T. & Ding, H. (2019). *Nat. Rev. Phys.* **1**, 609–626.

[bb41] Madey, J. M. (1971). *J. Appl. Phys.* **42**, 1906–1913.

[bb42] McMillan, E. M. (1945). *Phys. Rev.* **68**, 143–144.

[bb43] Nakamura, N. (2012). *Proceedings of the 3rd International Particle Accelerator Conference (IPAC’12)*, 20–25 May 2012, New Orleans, Louisiana, USA, pp. 1040–1044. TUXB02.

[bb44] Nodvick, J. S. & Saxon, D. S. (1954). *Phys. Rev.* **96**, 180–184.

[bb45] Pan, Z. (2020). *Research on optimization and design of advanced laser-driving storage ring.* PhD thesis, Tsinghua University, Beijing, China.

[bb46] Pan, Z., Rui, T., Wan, W., Chao, A., Deng, X., Zhang, Y., Huang, W. & Tang, C. (2019). *In Proceedings of the 39th International Free Electron Laser Conference (FEL’19)*, Hamburg, Germany, pp. 700–703. THP055.

[bb47] Pellegrini, C., Marinelli, A. & Reiche, S. (2016). *Rev. Mod. Phys.* **88**, 015006.

[bb48] Ratner, D. F. & Chao, A. W. (2010). *Phys. Rev. Lett.* **105**, 154801. 10.1103/PhysRevLett.105.154801 21230912

[bb49] Saldin, E., Schneidmiller, E. & Yurkov, M. (1998). *Opt. Commun.* **148**, 383–403.

[bb50] Saldin, E. L., Schneidmiller, E. A. & Yurkov, M. V. (2005). *Nucl. Instrum. Methods Phys. Res. A*, **539**, 499–526.

[bb51] Schwinger, J. (1949). *Phys. Rev.* **75**, 1912–1925.

[bb52] Schwinger, J. (1996). *A Quantum Legacy: Seminal Papers of Julian Schwinger*, pp. 307–331. Singapore: World Scientific.

[bb53] Stupakov, G. (2009). *Phys. Rev. Lett.* **102**, 074801. 10.1103/PhysRevLett.102.074801 19257677

[bb54] Tamai, A., Meevasana, W., King, P. D. C., Nicholson, C. W., de la Torre, A., Rozbicki, E. & Baumberger, F. (2013). *Phys. Rev. B*, **87**, 075113.

[bb55] Tang, C., Deng, X., Chao, A., Huang, W., Rui, T., Feikes, J., Li, J., Ries, M., Hoehl, A., Ratner, D., Granados, E., Feng, C., Jiang, B. & Wang, X. (2018). *Proceedings of the 60th ICFA Advanced Beam Dynamics Workshop on Future Light Sources (FLS’18)*, Shanghai, China, pp. 166–170. THP2WB02.

[bb56] Tang, C.-X. & Deng, X.-J. (2022). *Acta Phys. Sin.* **71**, 152901.

[bb57] Teng, L. (1984). *Minimizing the emittance in designing the lattice of an electron storage ring.* Technical Report. Fermi National Accelerator Laboratory, Batavia, IL, USA.

[bb58] Tsai, C.-Y. (2022*a*). *arXiv*:2205.15801.

[bb59] Tsai, C.-Y. (2022*b*). *Phys. Rev. Accel. Beams*, **25**, 064401.

[bb60] Tsai, C.-Y., Chao, A. W., Jiao, Y., Luo, H.-W., Ying, M. & Zhou, Q. (2021). *Phys. Rev. Accel. Beams*, **24**, 114401.

[bb61] Tzu, H. Y. (1948). *Proc. R. Soc. London A*, **192**, 231–246.

[bb62] Veksler, V. I. (1944). *Dokl. Akad. Nauk. USSR*, **43**, 346–348.

[bb63] Yu, L. H. (1991). *Phys. Rev. A*, **44**, 5178–5193. 10.1103/physreva.44.5178 9906572

[bb64] Zhang, Y. (2022). *Research on Longitudinal Strong Focusing SSMB Ring*, PhD thesis, Tsinghua University, Beijing, China.

[bb65] Zhang, Y., Deng, X. J., Pan, Z. L., Li, Z. Z., Zhou, K. S., Huang, W. H., Li, R. K., Tang, C. X. & Chao, A. W. (2021). *Phys. Rev. Accel. Beams*, **24**, 090701.

[bb66] Zhao, Z. (2010). *Rev. Accl. Sci. Tech.* **03**, 57–76.

[bb67] Zhao, Z., Wang, Z., Feng, C., Chen, S. & Cao, L. (2021). *Sci. Rep.* **11**, 23875. 10.1038/s41598-021-03354-0 PMC8668884 34903791

[bb68] Zhou, X., Wannberg, B., Yang, W., Brouet, V., Sun, Z., Douglas, J., Dessau, D., Hussain, Z. & Shen, Z.-X. (2005). *J. Electron Spectrosc. Relat. Phenom.* **142**, 27–38.

[bb69] Zhu, Z., Zhao, Z., Wang, D., Liu, Z., Li, R., Yin, L. & Yang, Z. (2017). *Proceedings of the 38th International Free Electron Laser Conference (FEL2017)*, 20–25 August 2017, Santa Fe, NM, USA, pp. 182–184. MOP055.

